# Effect of La and Si additives in Zr-doped HfO_2_ capacitors for pseudo-linear high-κ dielectric applications

**DOI:** 10.1186/s40580-025-00477-2

**Published:** 2025-03-06

**Authors:** Minjong Lee, Yong Chan Jung, Jin-Hyun Kim, Dushyant M. Narayan, Sehun Kang, Woo Young Park, Kivin Im, Jiyoung Kim

**Affiliations:** 1 Department of Electrical and Computer Engineering, The University of Texas at Dallas, Richardson, TX 75080 USA; 2 Department of Materials Science and Engineering, The University of Texas at Dallas, Richardson, TX 75080 USA; 3 R&D Division, SK Hynix Inc, Icheon, 17336 Republic of Korea

**Keywords:** High-κ, Low leakage current, DRAM, BEOL compatibility, Pseudo-linear dielectric, Hf_1–x_Zr_x_O_2_, La doping, Si doping, Phase transformation, Anti-ferroelectric

## Abstract

This study investigates the impact of dopants on Hf_1–x_Zr_x_O_2_-based capacitors for high-performance, hysteresis-free dielectric applications. Control of the crystalline structure of Hf_1–x_Zr_x_O_2_ films is crucial for achieving superior dielectric properties. The tetragonal (t) phase of Hf_1–x_Zr_x_O_2_ exhibits anti-ferroelectric (AFE) characteristics and shows promise due to its high dielectric constant (κ). However, hysteresis behavior in polarization–voltage sweeps due to AFE behavior presents a significant challenge, primarily due to the high energy loss when implemented in dynamic random-access-memory (DRAM) applications. To achieve hysteresis-free operation, this study focuses on suppressing AFE switching within the DRAM voltage range through Si or La doping in Hf_1–x_Zr_x_O_2_ films. Introducing small amounts of Si or La (< 1%) into Hf_1–x_Zr_x_O_2_ capacitors effectively diminishes AFE switching by influencing which structural phases are favored: Si doping tends to favor the amorphous phase, while La doping promotes the formation of the t-phase. La doping shows particular promise in enhancing pseudo-linear dielectric performance. ~ 0.9% La-doped Hf_0.25_Zr_0.75_O_2_ capacitors exhibit a markedly improved equivalent oxide thickness (EOT) of ~ 4.8 Å and a reduced leakage current density (J_leak_) of ~ 10^–7^ A/cm^2^ at 1 V, achieved at back-end-of-line (BEOL) compatible temperatures (< 400 °C). These results demonstrate a promising strategy for advancing energy-efficient high-κ dielectric materials in next-generation memory devices, offering a balanced combination of high capacitance, low leakage current, and BEOL compatibility.

**Graphical Abstract:**

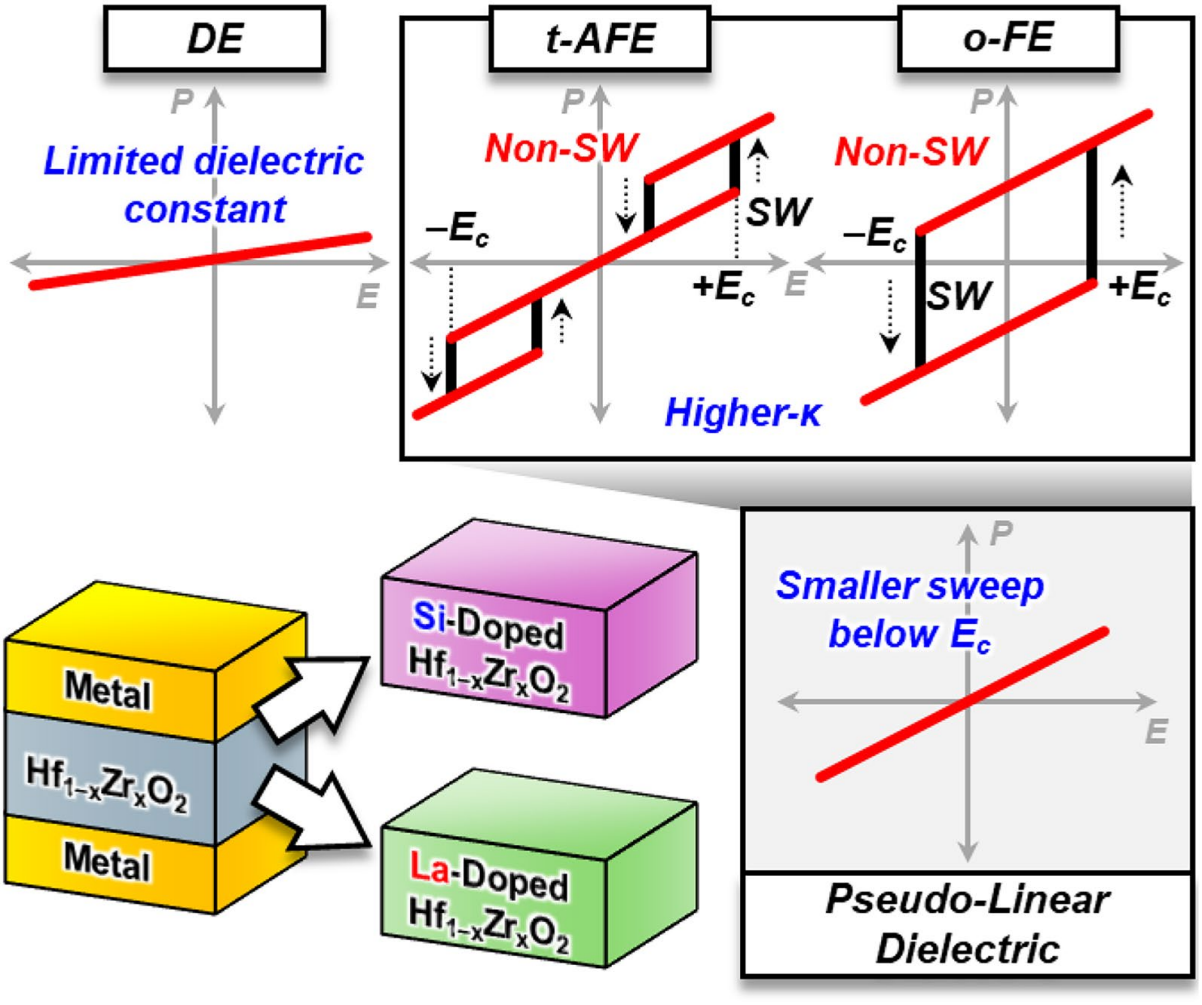

**Supplementary Information:**

The online version contains supplementary material available at 10.1186/s40580-025-00477-2.

## Introduction

The growing demand for high-bandwidth memory (HBM) is driving the evolution of dynamic random-access-memory (DRAM) technologies towards higher integration density and faster operation. A key challenge facing DRAM scaling is the need to achieve sufficiently high capacitance with scaled equivalent oxide thickness (EOT) while maintaining low leakage current [1]. Low processing temperature is also crucial for back-end-of-line (BEOL) process-based embedded DRAM (eDRAM) technologies. These requirements drive the search for BEOL-compatible high dielectric constant (κ) materials for eDRAM capacitor cells.

While narrow bandgap materials such as SrTiO_3_ (STO), BaSrTiO_3_ (BSTO), and TiO_2_ offer very high κ values exceeding 100, they typically suffer from high leakage current density (J_leak_) and are incompatible with complementary metal–oxide–semiconductor (CMOS) technology [2–5]. This trade-off between J_leak_ and κ poses a significant challenge in DRAM operation, as high J_leak_ leads to rapid loss of memory information in charge-based storage systems.

To address these challenges, one promising approach is to boost κ values in large bandgap dielectric materials. HfO_2_ films, with their large bandgap (> 5.1 eV) [6], have been of particular interest for high-κ dielectric applications in DRAM capacitors. However, HfO_2_ dielectrics are prone to relatively high J_leak_ due to oxygen vacancy formation, which creates charge trapping sites [7]. To address this issue, various dopants such as Al, Zr, and La have been explored, aiming to achieve high-κ (*i.e.*, low EOT) and low-J_leak_ simultaneously [7–9]. Doping also enables control over the crystalline structure of HfO_2_, as doped-HfO_2_ can exist in monoclinic (m), cubic (c), tetragonal (t), and orthorhombic (o) phases. These crystalline structures exhibit different κ values. These κ values decrease from t-phase showing the highest κ value, to o-phase, c-phase, and finally m-phase which displays a κ value of ~ 20. The theoretical value of κ in t-phase HfO_2_ is estimated to be ~ 70 [10, 11].

Notably, doped HfO_2_ materials typically form t-phase with anti-ferroelectric (AFE) characteristics and/or o-phase with ferroelectric (FE) characteristics after post-metal annealing (> 400 °C) [12, 13]. Among various dopants, Zr-doped HfO_2_ (Hf_1–x_Zr_x_O_2_) films have been extensively studied, with higher Zr compositions (x) showing AFE characteristics and increased κ values [14]. However, either FE or AFE phase in Hf_1–x_Zr_x_O_2_ materials leads to hysteresis behavior in polarization–voltage (P–V) and capacitance–voltage (C–V) loops, which causes large energy losses and reduced efficiency in energy storage applications like DRAM cells [15, 16]. To achieve useful energy storage capabilities for DRAM capacitors, a material with large capacitance and suppressed hysteresis behavior is therefore desired, thereby enhancing energy storage efficiency.

Our strategy for developing energy-efficient DRAM capacitors focuses on suppressing hysteresis behavior by applying an input voltage lower than the coercive voltage (V_c_) of the AFE Hf_1–x_Zr_x_O_2_ capacitors. To diminish hysteresis behavior, this study investigates the incorporation of La or Si dopants into Hf_1–x_Zr_x_O_2_ films, wherein they preferentially induce the formation of specific crystalline phases. These approaches provide functional design guidelines for achieving low EOT while maintaining low J_leak_ and hysteresis-free behavior.

## Methods/experimental

### Device fabrications

TiN/Hf_1–x_Zr_x_O_2_/TiN (90/7.5/90 nm) stack samples were fabricated on thermally grown 300 nm-thick SiO_2_/p-Si substrates. A blanket TiN bottom electrode (BE) was deposited by radio frequency (RF) magnetron sputtering at room temperature (RT). To deposit Hf_1–x_Zr_x_O_2_ layers, atomic layer deposition (ALD) processes were performed at 250 °C, using tetrakis(dimethylamido)hafnium (TDMAH) and tetrakis(dimethylamido)zirconium (TDMAZ) as Hf and Zr precursors, and O_3_ as the oxidant. For additional La and Si doping, we employed multi-dosing and super-cycle methods during the ALD cycles to uniformly introduce a small concentration of each dopant, using tris(N,N'-di-i-propylformamidinato)lanthanum (La(iPrfAMD)_3_) and Tris(dimethylamino)silane (TDMAS) as La and Si precursors, respectively. The La and Si doping concentrations in Hf_1–x_Zr_x_O_2_ films were estimated by the growth per cycle (GPC) characteristics of each oxide and/or the extrapolation of the X-ray photoelectron spectroscopy (XPS) profiles. The GPC values for HfO_2_, ZrO_2_, SiO_2_, and La_2_O_3_ are investigated as 1.0, 1.0, 0.3, and 0.8 Å/cy, respectively. To achieve a 0.9% La doping concentration in Hf_0.25_Zr_0.75_O_2_ films, an ALD super-cycle with a Hf:Zr:La ratio of 4:12:1 was used, resulting in a GPC of 1.68 nm per super-cycle. For films with varying thicknesses, different super-cycle numbers were applied, using 5, 4, and 3 cycles of Hf:Zr:La in a 4:12:1 ratio.

The (un)doped-Hf_1–x_Zr_x_O_2_ films were then immediately capped with sputtered TiN top electrodes (TE). Subsequently, a post-metal annealing process was conducted at 400 °C or 500 °C using rapid thermal annealing (RTA) for 1 min. After the annealing process, photolithography- and etching-based patterning were carried out to shape the circular TiN TE. The area of the TiN TE was calibrated by linear extrapolation of the square root of capacitance versus the device diameter to account for TE over-etching. All devices were subjected to measurement after 10^5^ bipolar cycles at an electric field of ± 2.5 MV/cm. This fabrication flow is consistent with our previous work [17, 18].

### Electrical characterization

The electrical characteristics of the Hf_1–x_Zr_x_O_2_ capacitors were measured using a Keithley 4200-SCS parameter analyzer for P–V and J–V characteristics, and an Agilent 4284A LCR meter for C–V characteristics. For the P–V measurements, we applied a triangle-shaped bipolar pulse with a rise/fall time of 10 µs. For the C–V measurements, we applied a 10 kHz small-signal input with an amplitude of 50 mV.

## Results and discussion

### Hysteresis-free high-κ dielectric operation

**Fig. 1:**
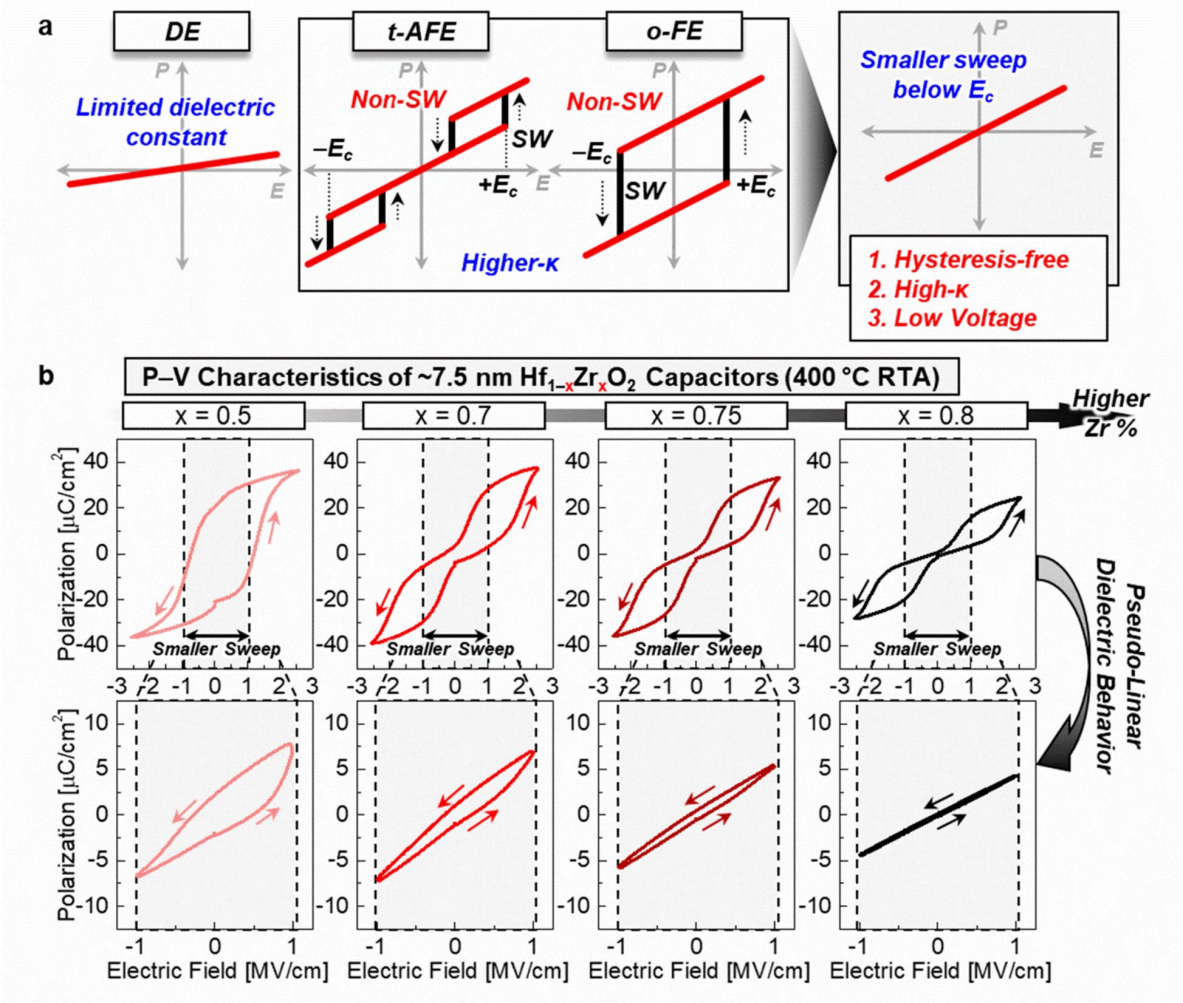
**a** Conceptual schematics for achieving hysteresis-free dielectric performance in anti-ferroelectric (AFE) or ferroelectric (FE) Hf_1–x_Zr_x_O_2_ capacitors. Applying voltage below DRAM operating range can leverage high-κ and low-J_leak_ properties without hysteresis behavior. **b** Polarization–electric field (P–E) characteristics, demonstrating the transition from FE to AFE behavior in Hf_1–x_Zr_x_O_2_ capacitors with increasing Zr content. A small range of voltage sweep can generate suppressed hysteresis in P–E characteristics

The key technique to achieve pseudo-linear dielectric operation with high-κ properties involves designing Hf_1–x_Zr_x_O_2_ capacitors with AFE characteristics that have a V_c_ (or coercive field, E_c_) greater than the DRAM operation voltage (typically ~ 1 V) (Fig. 1a). By elevating V_c_ (or E_c_) and applying a small sweep range of input voltages to these devices, hysteresis-free operation can be achieved while maintaining high-κ properties. Figure 1b illustrates the P–E characteristics of Hf_1–x_Zr_x_O_2_ capacitors with varying Zr compositions (x = 0.5, 0.7, 0.75, and 0.8). As Zr content increases, a phase transition from FE to AFE is observed in P–E measurements using electric field cycling at ± 2.5 MV/cm. A small electric field sweep of 1 MV/cm during P–E measurements results in diminished hysteresis behavior with increasing Zr composition, due to the relatively higher E_c_ [14, 19].

**Fig. 2:**
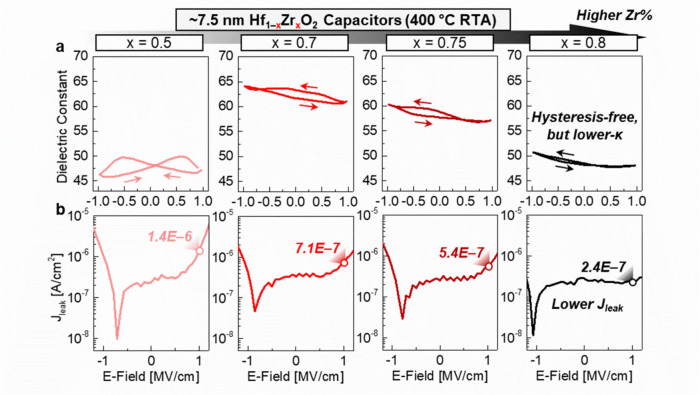
**a** Capacitance–electric field (C–E) characteristics-based dielectric constant (κ) performances in Hf_1–x_Zr_x_O_2_ capacitors with increasing Zr content. Increased Zr contents can generate hysteresis-free operation, but lower κ values. **b** Current density–electric field (J–E) characteristics of Hf_1–x_Zr_x_O_2_ capacitors. Higher Zr compositions result in reduced leakage current density (J_leak_)

The suppressed hysteresis behavior in Hf_1–x_Zr_x_O_2_ capacitors is evident in 10 kHz C–E measurements (Fig. 2a). At 0 V, the κ values are 47.8, 63.3, 58.8, and 48.3 for Hf_1–x_Zr_x_O_2_ capacitors with Zr compositions of x = 0.5, 0.7, 0.75, and 0.8, respectively. Devices in the region of x = 0.7–0.75 exhibit the highest κ values, consistent with previous reports [8, 20]. This trend in κ values correlates with the morphotropic phase boundary (MPB) between o- and t-phases in Hf_1–x_Zr_x_O_2_ films, with the κ values peaking near x =  ~ 0.7. Additionally, J_leak_ decreases with higher Zr composition, reducing from 1.4 × 10^–6^ A/cm^2^ to 2.4 × 10^–7^ A/cm^2^ at 1 MV/cm as Zr composition increases from 0.5 to 0.8 (Fig. 2b).

Despite the reduction in J_leak_ with higher Zr composition, continuously increasing Zr content results in a lower κ value. Moreover, even in the composition range of x = 0.7–0.75 which is optimal for high-κ properties, some degree of hysteresis behavior persists. This residual hysteresis behavior underscores the limitations of relying solely on composition control to optimize Hf_1–x_Zr_x_O_2_ capacitors. We thus investigated additional strategies, such as the incorporation of other additives, to further enhance the dielectric performance for DRAM applications.

### Si doping for the promotion of the amorphous phase

**Fig. 3:**
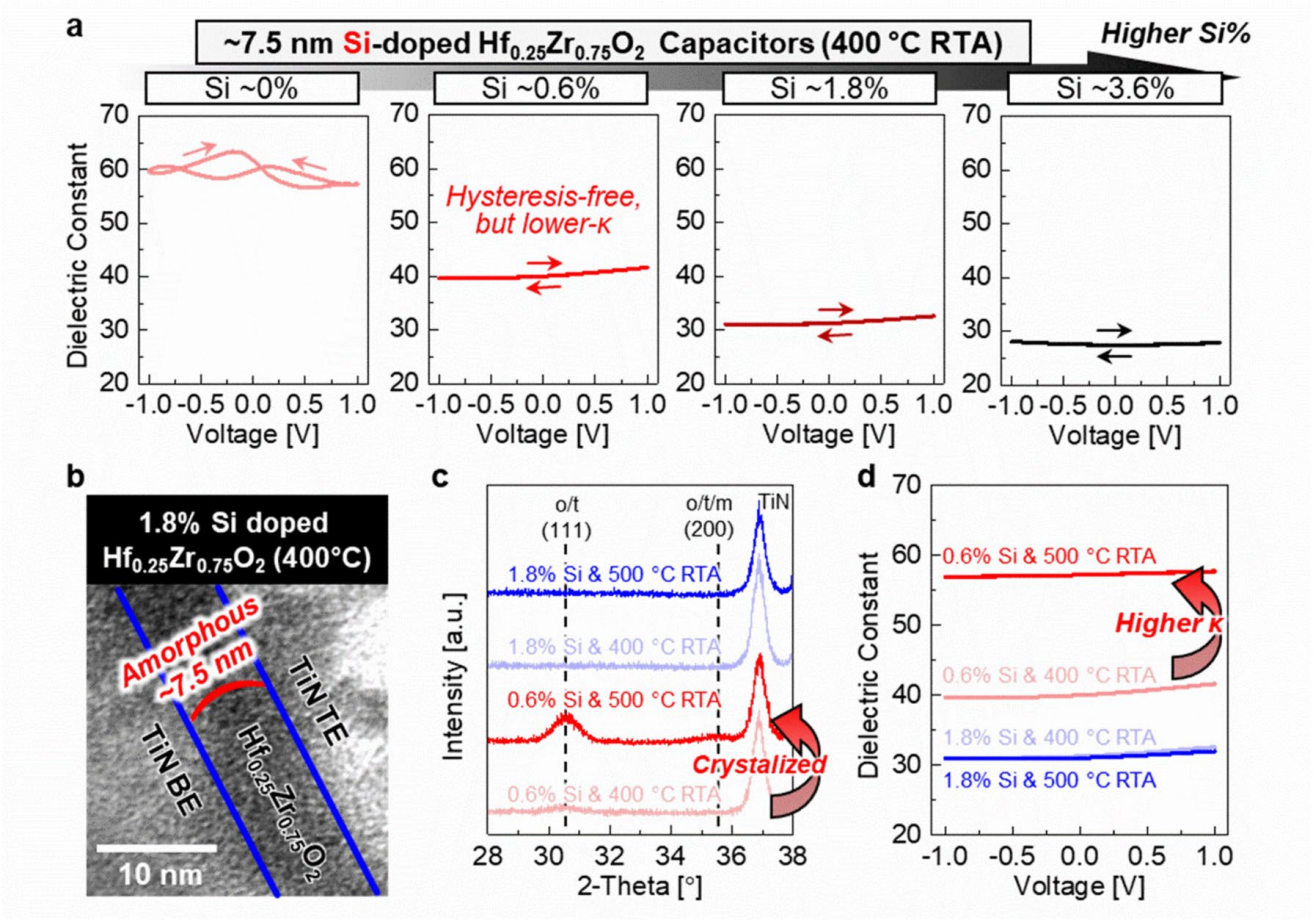
**a** C–V characteristics-based κ performances in Si-doped Hf_1–x_Zr_x_O_2_ capacitors with increasing Si contents. Hysteresis-free behavior is observed with ~ 0.6% Si doping, but degraded κ values. **b** Transmission electron microscopy (TEM) images confirming the amorphous states of 0.6% Si-doped Hf_0.25_Zr_0.75_O_2_ capacitors. These amorphous states lead to decreased κ values. **c** Grazing incidence X-ray diffraction (GIXRD) analysis of 0.6% and 1.8% Si-doped Hf_0.25_Zr_0.75_O_2_ capacitors, annealed at 400 °C and 500 °C using rapid thermal annealing (RTA). The 0.6% Si-doped Hf_0.25_Zr_0.75_O_2_ film crystallizes at 500 °C RTA. **d** C–V characteristics of 0.6% and 1.8% Si-doped Hf_0.25_Zr_0.75_O_2_ capacitors, annealed at 400 °C and 500 °C RTA. Increased κ values are demonstrated in 0.6% Si-doped Hf_0.25_Zr_0.75_O_2_ capacitors after 500 °C annealing

In addition to Zr composition control, we extended our studies to the introduction of Si into the Hf_0.25_Zr_0.75_O_2_ thin films. Incorporation of Si as a dopant at a concentration greater than 0.6% into Hf_0.25_Zr_0.75_O_2_ films results in hysteresis-free behavior in the C–V characteristics within a 1 V bipolar operating window, as shown in Fig. 3a. However, the κ values decrease with increasing Si-doping. The κ values at 0 V are 59.9, 40.0, 31.3, and 27.4 for Si-doped Hf_1–x_Zr_x_O_2_ capacitors with Si concentration of 0%, 0.6%, 1.8%, and 3.6%, respectively. This phenomenon is attributed to the fact that Hf_0.25_Zr_0.75_O_2_ films have a high amorphous phase fraction upon application of RTA at 400 °C even with a slight addition of Si dopants at a concentration of 0.6%. Additionally, at higher Si concentration, SiO_2_ can form within the Hf_0.25_Zr_0.75_O_2_ films, which lowers the effective κ values. Figure 3b shows transmission electron microscopy (TEM) images confirming the amorphous state of 0.6% Si-doped Hf_0.25_Zr_0.75_O_2_ capacitors after annealing at 400 °C. These observations are consistent with previous reports indicating that Si-doped HfO_2_ generally requires annealing temperatures exceeding 600 °C to facilitate the formation of o- or t-phase crystalline phases [13, 21, 22].

To leverage the phases which exhibit high-κ properties, it becomes necessary to increase the annealing temperature for Si-doped Hf_0.25_Zr_0.75_O_2_ capacitors. Figure 3c presents grazing incidence X-ray diffraction (GIXRD) analysis for 0.6% and 1.8% Si-doped Hf_0.25_Zr_0.75_O_2_ films annealed at 400 °C and 500 °C. For these measurements, the blanket TiN TE of each sample was removed using SC-1 (NH_4_OH:H_2_O_2_ of 1:1) after post-metal annealing processes. Although it is difficult to distinguish between o-, t-, and m-phases in GIXRD spectra, we confirm that annealing at 500 °C induces crystallization in 0.6% Si-doped Hf_0.25_Zr_0.75_O_2_ films, evidenced by an increased o/t(1 1 1) peak centered at 2-theta of 30.6°. In contrast, the sample with 1.8% Si remains amorphous even after 500 °C RTA. The resulting crystallization of Si-doped Hf_0.25_Zr_0.75_O_2_ films at 500 °C RTA is reflected in the electrical characteristics of the capacitor devices (Fig. 3d). While devices with 1.8% Si maintain a consistent κ value of ~ 31 after both 400 °C and 500 °C RTA, those with 0.6% Si show improved κ values from 40.0 to 57.2 when increasing the RTA temperature from 400 °C to 500 °C.

In this context, Si doping can be employed to realize pseudo-linear dielectric behavior. While a comparably high κ value can be attained without hysteresis behavior using a slight Si doping of 0.6%, the required annealing temperature of 500 °C is not suitable for a BEOL-compatible process techniques. This limitation necessitates the exploration of alternative dopants for the promotion of high κ phase crystalization in Hf_1–x_Zr_x_O_2_ films while maintaining BEOL compatibility.

### La doping for the promotion of the tetragonal phase

**Fig. 4:**
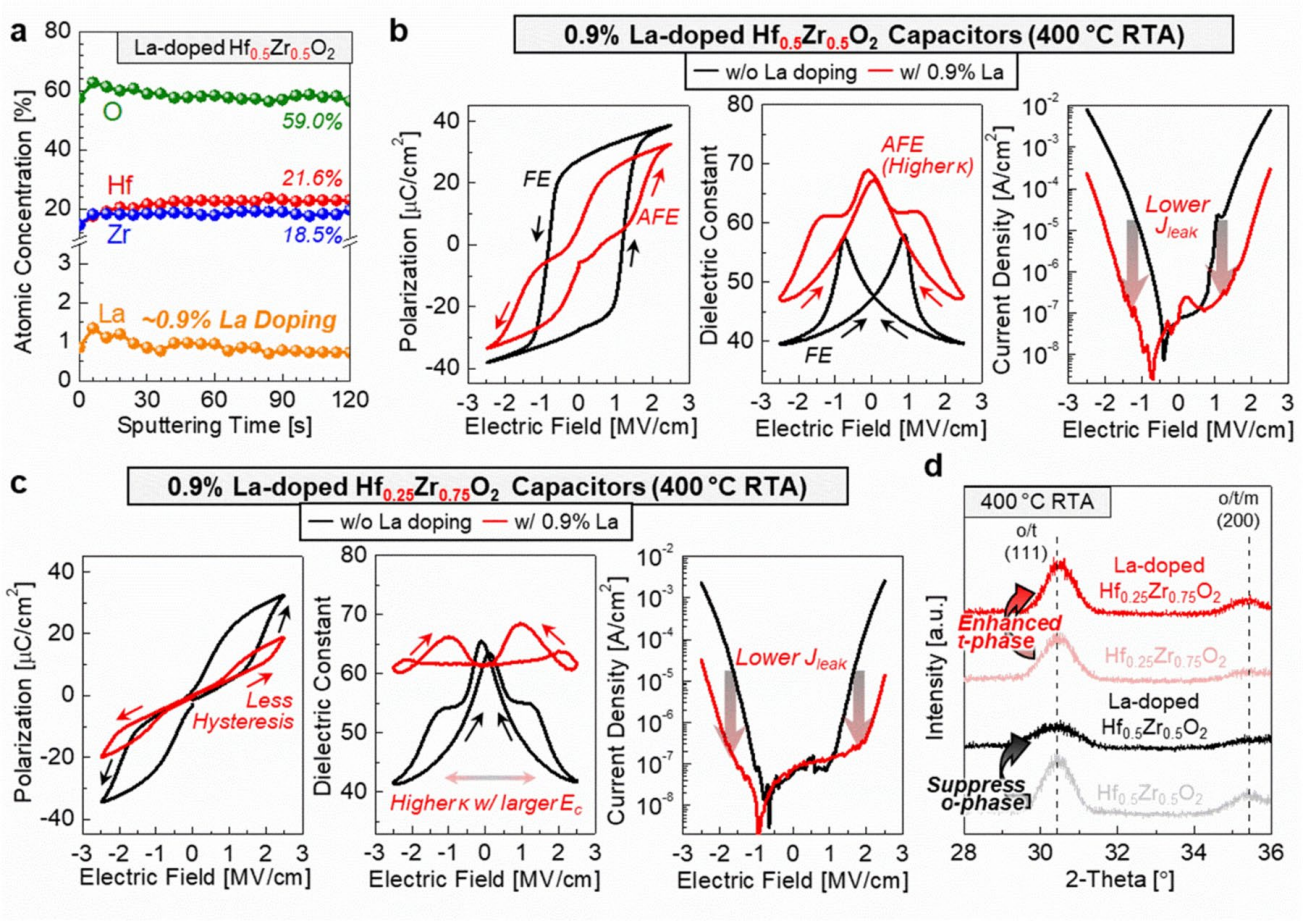
**a** Atomic concentration profile of La-doped Hf_0.5_Zr_0.5_O_2_ films, obtained through XPS analysis using Ar ion sputtering at 1 kV with 6 s intervals. **b** P–E, κ–E, and J–E characteristics of Hf_0.5_Zr_0.5_O_2_ capacitors with and without ~ 0.9% La doping. An FE to AFE transition is observed upon doping La into Hf_0.5_Zr_0.5_O_2_ capacitors, accompanied by decreased J_leak_. **c** P–E, κ–E, and J–E characteristics of Hf_0.25_Zr_0.75_O_2_ capacitors with and without ~ 0.9% La doping. Increased κ and decreased J_leak_ are observed upon doping La into Hf_0.25_Zr_0.75_O_2_ capacitors. **d** GIXRD analysis of 0.9% La-doped and undoped Hf_0.25_Zr_0.75_O_2_ and Hf_0.5_Zr_0.5_O_2_ films, annealed at 400 °C. An interesting phase transition to AFE t-phase is observed in Hf_1–x_Zr_x_O_2_ films

The impact of La doping was investigated in Hf_1–x_Zr_x_O_2_ capacitors as an alternative dopant candidate. X-ray photoelectron spectroscopy (XPS) analysis revealed that slight La doping in Hf_0.5_Zr_0.5_O_2_ films does not significantly alter the Hf/Zr and O compositions as compared to undoped samples (Fig. S1). Figure 4a shows the atomic concentration profile of La-doped Hf_0.5_Zr_0.5_O_2_ films, obtained through XPS analysis using Ar ion sputtering at 1 kV with 6 s intervals. The La content was estimated at ~ 0.9%, with an O/(Hf + Zr + La) ratio of 1.44, while undoped Hf_0.5_Zr_0.5_O_2_ films exhibited an O/(Hf + Zr) ratio of 1.56.

The effect of 0.9% La doping is particularly pronounced when considering electrical performance. Figure 4b shows the electrical characteristics of Hf_0.5_Zr_0.5_O_2_ capacitors with and without 0.9% La doping. A transition is observed upon La doping, where the FE behavior shifts to AFE behavior, as indicated by the P–V hysteresis curves. This transition to AFE behavior results in higher κ values compared to the FE state, with the κ value increasing from 48.1 to 68.5 at 0 V in La doped Hf_0.5_Zr_0.5_O_2_ capacitors. Additionally, La doping significantly reduces J_leak_, lowering it from 7.7 × 10^–3^ A/cm^2^ to 2.96 × 10^–4^ A/cm^2^. This can be attributed to reduced oxygen vacancies in La-doped Hf_1–x_Zr_x_O_2_ films [7, 23].

To reduce the hysteresis behavior further, we investigated the effect of La doping in AFE Hf_0.25_Zr_0.75_O_2_ capacitors (Fig. 4c). During the same bipolar operation with an electric field of ± 2.5 MV/cm, suppressed hysteresis behavior was observed in La doped Hf_0.25_Zr_0.75_O_2_ capacitors, indicating that the E_c_ shifted to higher values as compared to undoped Hf_0.25_Zr_0.75_O_2_ capacitors. This effect is clearly seen in the C–V characteristics, where a higher κ and E_c_ are observed with 0.9% La doping. To gain a deeper understanding of the improved dielectric performance resulting from La doping, we conducted GIXRD analysis on both Hf_0.25_Zr_0.75_O_2_ and Hf_0.5_Zr_0.5_O_2_ films, with and without 0.9% La doping (Fig. 4d). An interesting effect of La doping was observed: La doping in Hf_0.5_Zr_0.5_O_2_ films led to reduced o-phase formation, which may explain the transition from FE electrical behavior to AFE electrical behavior. Furthermore, in Hf_0.25_Zr_0.75_O_2_ films which ordinarily exhibit AFE electrical behavior, we observe an enhancement of the t-phase upon La doping. This suggests that slight La doping into Hf_1–x_Zr_x_O_2_ films plays a role in stabilizing the t-phase to a greater extent.

The combined electrical and crystal structure analyses highlight the effect of La doping in Hf_1–x_Zr_x_O_2_ films as a source of additional stress that promotes the formation of the AFE t-phase, which exhibits improved high-κ and low-J_leak_ properties. Notably, the annealing temperature required for crystallization is below 400 °C upon La doping, making it a highly suitable option for BEOL-compatible capacitors (see Fig. S2 for the durability characteristics of 0.9% La-doped Hf_0.25_Zr_0.75_O_2_ capacitors, further demonstrating their potential suitability for DRAM device applications).

**Fig. 5:**
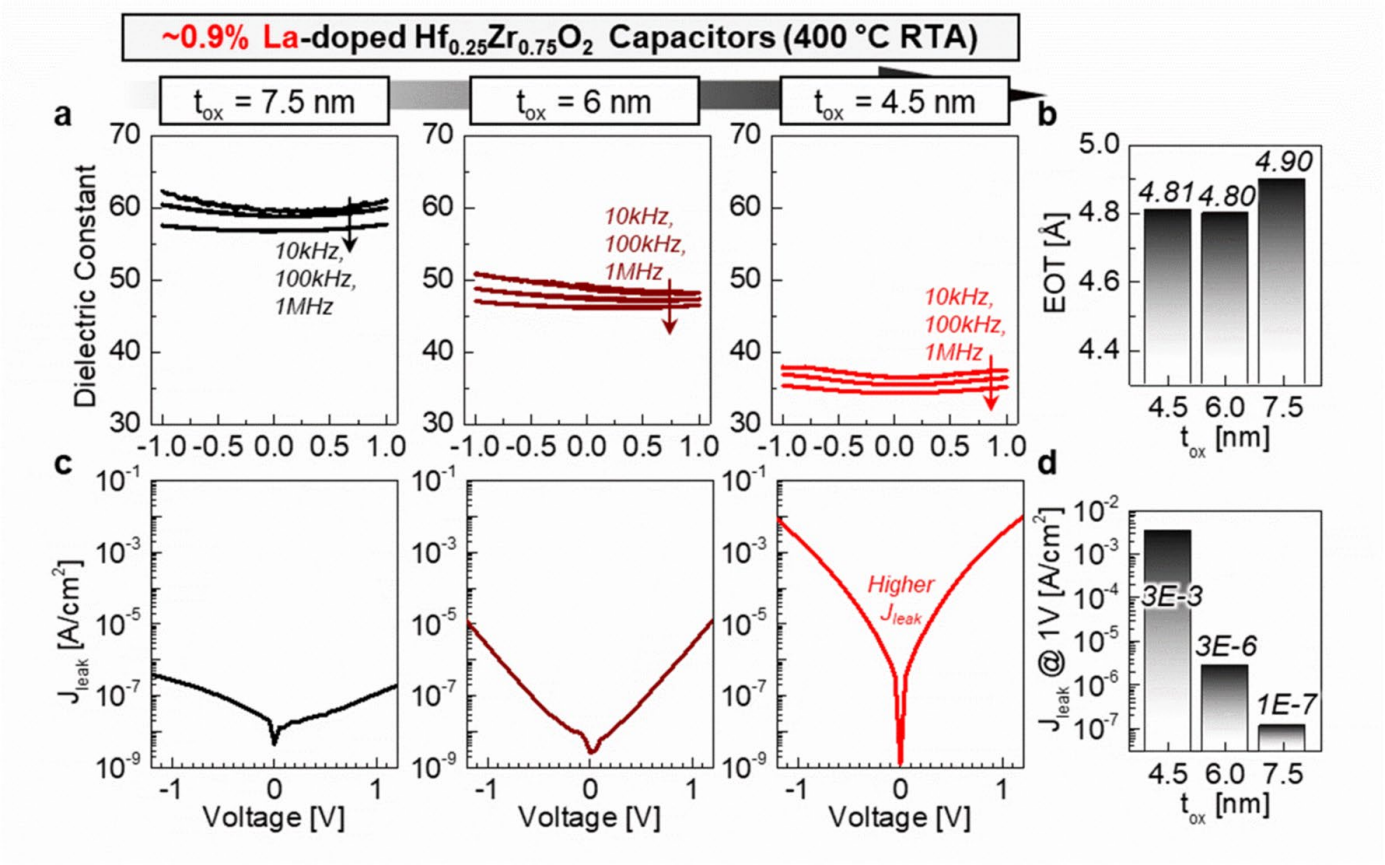
**a** Voltage-dependent κ properties in ~ 0.9% La-doped Hf_0.25_Zr_0.75_O_2_ capacitors with thicknesses scaling from 7.5 nm to 4.5 nm. **b** Estimated equivalent oxide thickness (EOT) values for each device. **c** J–V characteristics of ~ 0.9% La-doped Hf_0.25_Zr_0.75_O_2_ capacitors with thicknesses scaling from 7.5 nm to 4.5 nm. **d** J_leak_ at 1 V for each device

We also performed thickness (t_ox_) scaling studies on the 0.9% La-doped Hf_0.25_Zr_0.75_O_2_ capacitors, analyzing film thickness ranging from 7.5 nm to 4.5 nm. Figure 5a shows the voltage-dependent κ properties of the devices, obtained at small-signal frequencies of 10 kHz, 100 kHz, and 1 MHz. Reduced hysteresis was observed within an input voltage of ± 1 V operation. The κ values were measured to be 59.7, 49.3, and 36.5 at 0 V for t_ox_ of 7.5 nm, 6 nm, and 4.5 nm in 10 kHz C–V measurements, respectively. The frequency of 10 kHz and above are sufficient to suppress ionic or interfacial contributions that lead to slow polarization responses; however, this universal dielectric response is essential for further studies on the significant contributors of defects to dielectric behavior. The EOT values were estimated using the equation of EOT = (3.9 × t_ox_)/κ: the results show that the EOT is ~ 4.8–4.9 Å across the thickness range (Fig. 5b). An interesting performance is observed in the 4.5 nm-thick 0.9% La-doped Hf_0.25_Zr_0.75_O_2_ films, where the P–E characteristics with ± 2.5 MV/cm exhibit paraelectric behavior despite showing a low EOT value (Fig. S3a). The lack of crystallization in this ultra-thin film is attributed to the increased surface energy when scaling down the Hf_1–x_Zr_x_O_2_ thickness, which requires a higher annealing temperature for crystallization [24]. We believe the comparable EOT value in 4.5 nm-thick films are due to their increased susceptibility to the roughness of the bottom electrode, which can reduce the effective thickness. The root mean square (RMS) value of roughness of our sputtered TiN bottom electrode is ~ 1.33 nm, which accounts for around 30% of the 4.5 nm Hf_1–x_Zr_x_O_2_ films (Fig. S3b). This roughness thus can result in a higher electric field than expected, leading to lower equivalent oxide thickness (EOT) values.

Figure 5c shows the J–V characteristics of the 0.9% La-doped Hf_0.25_Zr_0.75_O_2_ capacitors with thicknesses scaling from 7.5 nm to 4.5 nm. The J_leak_ at 1 V is measured as 1 × 10^–7^ A/cm^2^, 3 × 10^–6^ A/cm^2^, and 3 × 10^–3^ A/cm^2^ respectively for t_ox_ of 7.5 nm, 6 nm, and 4.5 nm (Fig. 5d). This study thus represents significant progress in high-κ dielectric engineering, demonstrating that the slight La doping in Hf_1–x_Zr_x_O_2_ films leads to outstanding EOT, and J_leak_ without hysteresis, as compared with previous reports [2, 24–31]. The suppressed hysteresis by doping with 0.9% La results in a high energy efficiency (U_efficiency_) of 96%, compared to 86% for undoped Hf_0.25_Zr_0.75_O_2_ capacitors (Fig. S4). The BEOL compatible process temperatures also offers easy integration into existing process flows, reducing the complexity for implementation in eDRAM applications.

## Conclusions

**Fig. 6:**
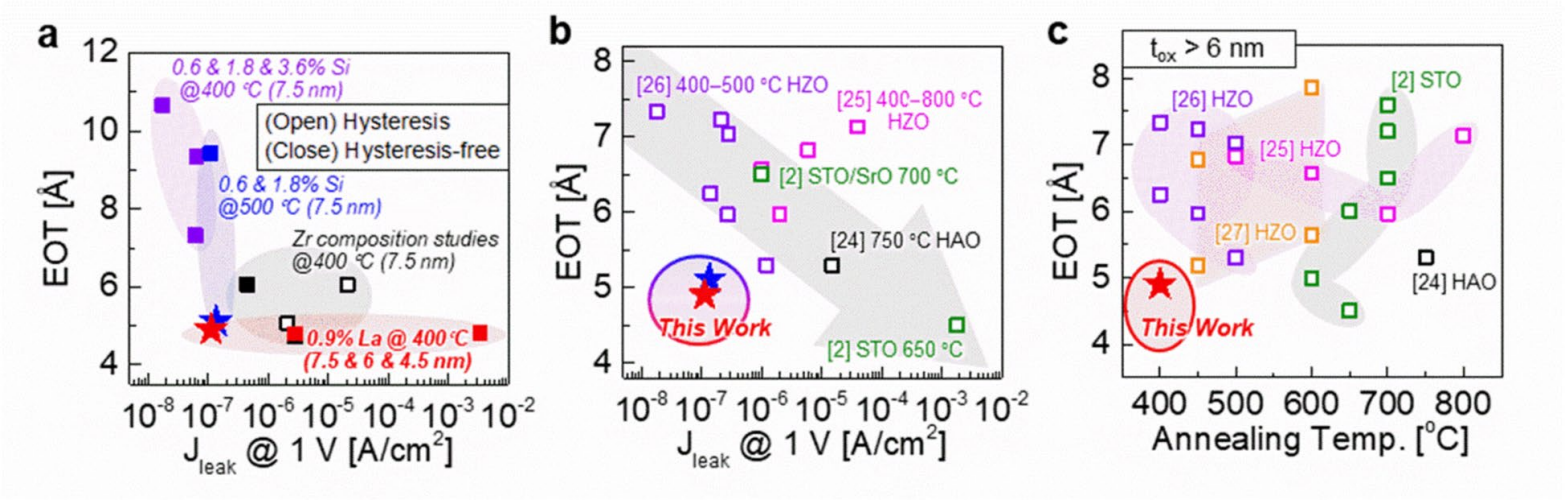
**a** Summary of our results for (un)doped-Hf_1–x_Zr_x_O_2_ capacitors with La and Si doping. **b** Benchmark comparison of κ versus J_leak_ at 1 V against previous records. **c** Benchmark comparison of EOT versus annealing temperature against previous records. The resulting properties demonstrate excellent dielectric performances at BEOL-compatible temperatures

This study demonstrated the high-κ and low-J_leak_ dielectric performance of pseudo-linear Hf_1–x_Zr_x_O_2_ capacitors, achieved thorugh Zr composition control as well as Si or La doping (Fig. 6a). We identified a significant limitation in relying solely on engineering Zr composition (x) to simultaneously achieve hysteresis-free, low EOT, and low J_leak_ properties. The introduction of Si or La as dopants thereby proved to be beneficial in achieving high-performance dielectric behavior through control of the crystallization of specific structural phases. The effect of Si doping on Hf_1–x_Zr_x_O_2_ films was confirmed to suppress crystallization which enhanced hysteresis-free behavior. However, this required higher annealing temperatures of 500 °C to leverage the high-κ properties of the crystalline phases. In contrast, slight La doping favors crystallization in the t-phase, leading to high E_c_ which is consistent with AFE behavior. This dopant resulted in higher-κ and lower-J_leak_ properties at a lower annealing temperature of 400 °C. The dielectric performance for 0.9% La-doped Hf_0.25_Zr_0.75_O_2_ capacitors are excellent from the perspective of EOT (~ 4.9 Å), J_leak_ (~ 10^–7^ A/cm^2^), and annealing temperature (400 °C) (Fig. 6b, c) (see Fig. S5 for additional benchmarking comparison). These findings not only highlight the remarkable potential of additives in Hf_1–x_Zr_x_O_2_ films to achieve customizable dielectric properties, but also marks a significant milestone in phase engineering towards high-performance dielectrics for DRAM applications.

## Supplementary Information

Additional file 1.

## Data Availability

The datasets used and/or analyzed during the current study are available from the corresponding author on reasonable request.
