# Supplementary material for: Effect of La and Si additives in Zr-doped HfO2 capacitors for pseudo-linear high-κ dielectric applications
Source: Nano Converg. 2025 Mar 6;12:15. doi: 10.1186/s40580-025-00477-2 (PMC11885729; doi:10.1186/s40580-025-00477-2)
Supplement: Supplementary file 1 — Additional file 1. [file 40580_2025_477_MOESM1_ESM.docx]

**Supporting Information**

**Effect of La and Si Additives in Zr-doped HfO_2_ Capacitors
for Pseudo-Linear High-κ Dielectric Applications**

Minjong Lee^1,†^, Yong Chan Jung^2,†^, Jin-Hyun Kim^2^, Dushyant M. Narayan^2^, Sehun Kang^3^,
Woo Young Park^3^, Kivin Im^3^, Jiyoung Kim^1,2,*^

^1^ Department of Electrical and Computer Engineering, The University of Texas at Dallas, Richardson, Texas 75080, USA.

^2^ Department of Materials Science and Engineering, The University of Texas at Dallas, Richardson, Texas 75080, USA.

^3^ R&D division, SK hynix Inc., Icheon 17336, Republic of Korea.

^†^ These authors contributed equally to this work.

^*^ E-mail address: jiyoung.kim@utdallas.edu

**Supporting Figure 1**


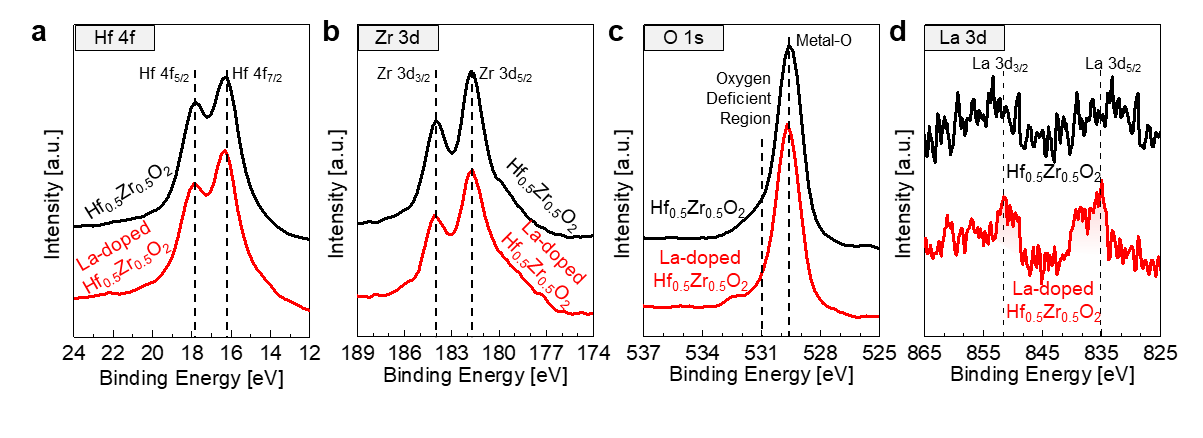


Figure S1. X-ray photoelectron spectroscopy (XPS) analysis of Hf_0.5_Zr_0.5_O_2_ films with and without La doping. The profiles of (a) Hf 4f, (b) Zr 3d, (c) O 1s, and (d) La 3d are shown.

**Supporting Figure 2**


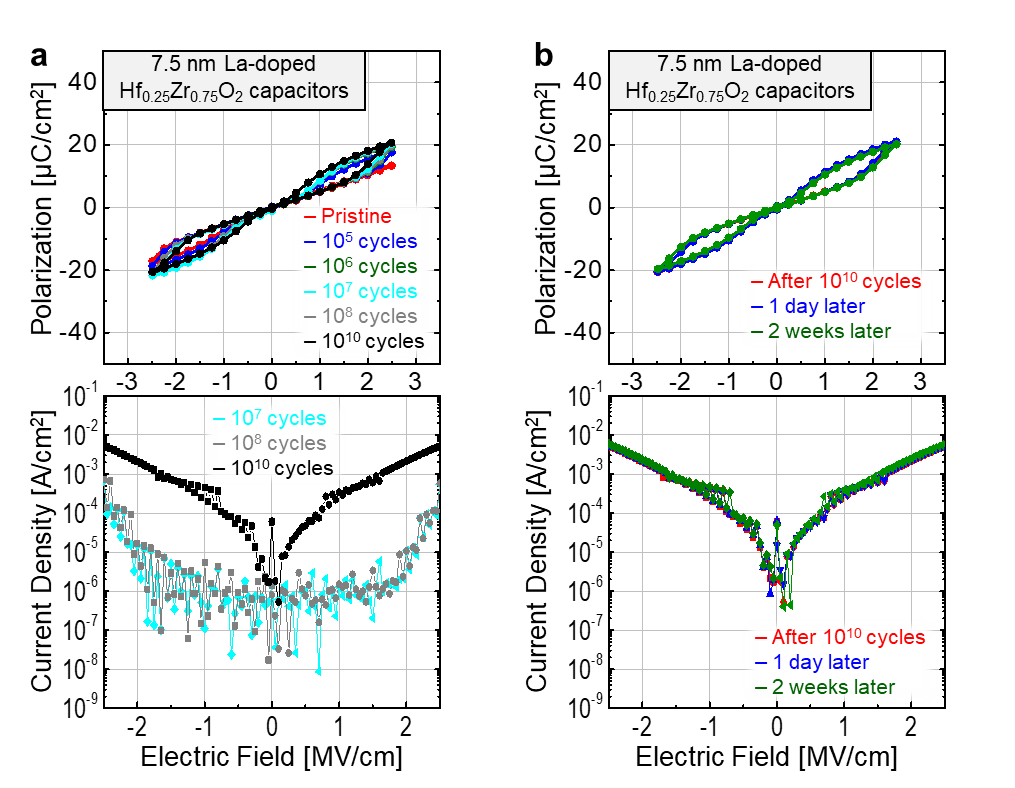


Figure S2. (a) Polarization–voltage (P–V) and current density–voltage (J–V) characteristics of 0.9% La-doped Hf_0.25_Zr_0.75_O_2_ capacitors after bipolar cycles at 50 kHz and ±2.5 MV/cm. (b) P–V and J–V characteristics after aging time of 1 day and 2 weeks.

**Supporting Figure 3**


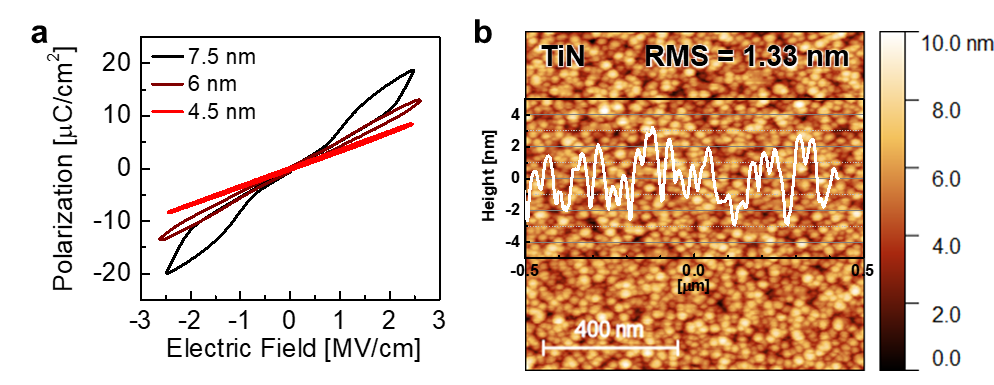


Figure S3. (a) P–V characteristics of 0.9% La-doped Hf_0.25_Zr_0.75_O_2_ capacitors with thickness scaling from 7.5 nm to 4.5 nm. (b) Atomic force microscopy (AFM) analysis of our TiN bottom electrodes, showing the RMS value of roughness is 1.33 nm.

**Supporting Figure 4**


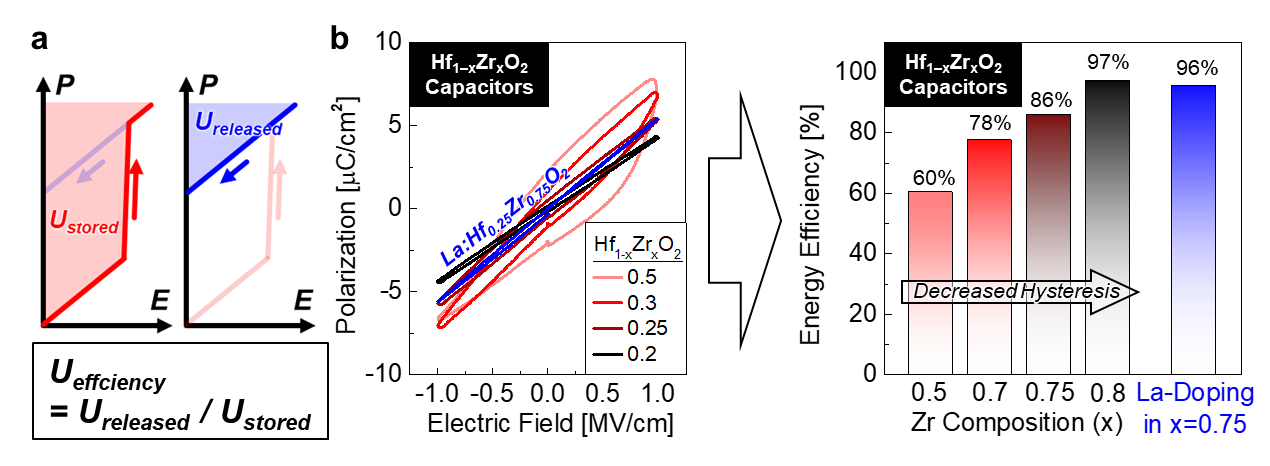


Figure S4. (a) Schematic illustration defining the three energy density components from P–E curves. (b) P–E characteristics and energy efficiency (U_efficiency_) of Hf_1–x_Zr_x_O_2_ devices with varying Zr compositions (x = 0.5, 0.7, 0.75, and 0.8), along with La-doped Hf_0.25_Zr_0.75_O_2_ capacitors.

**Supporting Figure 5**


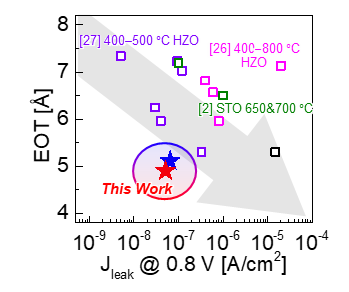


Figure S5. Benchmark comparison of κ versus J_leak_ at 0.8 V against previous records.
